# Antigen-specific downregulation of miR-150 in CD4 T cells promotes cell survival

**DOI:** 10.3389/fimmu.2023.1102403

**Published:** 2023-01-27

**Authors:** Antoine Ménoret, Federica Agliano, Timofey A. Karginov, Keaton S. Karlinsey, Beiyan Zhou, Anthony T. Vella

**Affiliations:** Department of Immunology, UConn Health, Farmington, CT, United States

**Keywords:** MiR-150, lymphocyte, non-coding RNA, superantigen, mTOR, apoptosis, mitochondria, superoxidedismutase

## Abstract

MicroRNA-150 (miR-150) has been shown to play a general role in the immune system, but very little is known about its role on CD4^+^ T cell responses. During T cell responses against superantigen Staphylococcal Enterotoxin A, miR-150 expression was down-regulated in antigen-specific CD4^+^ T cells but up-regulated in CD8^+^ T cells. CD4^+^ and CD8^+^ T cell clonal expansion was greater in miR-150-KO mice than in WT mice, but miR-150 selectively repressed IL-2 production in CD4^+^ T cells. Transcriptome analysis of CD4^+^ T cells demonstrated that apoptosis and mTOR pathways were highly enriched in the absence of miR-150. Mechanistic studies confirmed that miR-150 promoted apoptosis specifically in antigen-specific CD4^+^ T cells, but not in bystander CD4^+^ nor in CD8^+^ T cells. Furthermore, inhibition of mTOR-linked mitochondrial superoxidedismutase-2 increased apoptosis in miR-150^-/-^ antigen-specific CD4^+^ T. Thus, miR-150 impacts CD4^+^ T cell helper activity by attenuating IL-2 production along with clonal expansion, and suppresses superoxidedismutase to promote apoptosis.

## Introduction

T cell activation and clonal expansion play key roles in adaptive responses contributing to host defense and anti-tumor immunity. The extracellular signals responsible for initial T cell activation and further T cell clonal expansion have been well described. More recently, the field has learned a considerable amount about mechanisms contributing to these processes. For example, cell metabolic switches can fine-tune the intracellular signaling pathways to ensure proper T cell priming and expansion (1). Moreover, non-coding RNAs can actively regulate T cell activation by modulating multiple pathways (2, 3). Among non-coding RNAs, micro-RNAs (miRNAs) are a family of evolutionary conserved endogenous noncoding RNAs about 21-25nt in length that control posttranscriptional gene repression (3, 4). The key roles of miRNAs in T cells have also been demonstrated in mice defective for DICER, a non-redundant enzyme required for miRNA biogenesis (5). Individual miRNAs have been further explored in T cell ontogeny, activation, differentiation, and establishment of long-term memory (2, 3, 6, 7).

T cells responding to superantigens, such as Staphylococcal Enterotoxin A (SEA), can provide valuable information to understand pathogen-induced acute lung injury (ALI) (8, 9), systemic inflammatory response syndrome (SIRS) (10), sudden infant death syndrome (11), chronic sinusitis (12), and lethal toxic shock (13, 14). Moreover, SEA is a superantigen for its ability to directly linking MHC with specific Vβ regions of the TCR on CD4^+^ and CD8^+^ T cells concomitantly, driving massive cytokine production with robust T cells expansion for both lineages (15, 16). Therefore, this system offers a physiological way to simultaneously activate and follow endogenous oligoclonal CD4^+^ and CD8^+^ T cells using the same immunogen, providing built-in controls to study antigen and non-antigen-specific T cells in the same mouse without the need for adoptive T cell transfer.

Among all miRNAs, miR-150 is one of the most abundant miRNAs in both naïve CD4^+^ and CD8^+^ T cells and a crucial regulator for lymphopoiesis (3). In CD8^+^ T cells, studies have shown that miR150 negatively impact memory formation (6, 7, 17) and involves regulates several key apoptotic factors, like Bcl-2, BcL-X, and c-Myb, which have been identified as miR-150 targets in CD8^+^ T cells (7, 18).

Here, we report that downregulation of miR-150 in CD4^+^ T cell antigen-specific response is necessary to ensure cell survival. RNAseq analysis uncovers a critical role for miR-150 in apoptosis and the mTOR pathway, but only in activated, antigen-specific CD4^+^ T cells. In miR-150-KO antigen-specific CD4^+^ T cells, a higher mitochondrial membrane potential was measured and the mitochondrial Manganese-containing superoxide dismutase (SOD2) was shown to play a critical role in guarding against mitochondrial oxidative stress that is essential for protection against apoptosis. In total, this novel link of miR-150’s regulation of SOD2 to program activation-induced T cell death represents a new checkpoint for therapeutic regulation that could be targeted for preventing excessive CD4^+^ T cell activity.

## Methods

### Mice

C57BL/6 CD45.1+ mice (Stock #2014), 8 to 16 weeks males and females, were purchased from the Jackson Laboratory (Bar Harbor, ME). All mice were maintained in the central animal facility at the University of Connecticut Health (UCH) in accordance with federal guidelines. MiR-150-KO mice were provided by Dr. Beiyan Zhou, University of Connecticut Health. The present study was approved by the UCH’s Animal Care Committee.

### Reagents

*Staphylococcus* enterotoxin A (*S. aureus* enterotoxin A) was purchased from Toxin Technology Inc. (Sarasota, FL). Ionomycin was purchased from Life Technology (Grand Island, NY). Phorbol 12-Myristate 13-Acetate (PMA) and Brefeldin A (BFA) were purchased from EMD Millipore Corporation (Billerica, MA). Human rIL-2 was obtained from the NIH. Ruxolitinib (sc-364729) was purchased from Santa Cruz Biotechnology (Santa Cruz, CA). Lipopolysaccharide (LPS) (from *salmonella enterica typhimurium*) was purchased from Sigma Aldrich (St. Louis, MO). Live/Dead UV blue stain was purchased from Invitrogen (San Diego, CA). LIVE/DEAD Fixable Blue Dead^−^ was purchased from ThermoFischer, Catalog #L23105, (Waltham, MA).

### Antibodies, ELISA, and multiplex

Anti-IL-2 antibodies (clones S4B6-1 and JES6-1A12), anti-CD25 antibody (clone PC-61.5.3), and rat isotype controls were purchased from Bio X Cell (West Lebanon, NH); Anti-CD8^+^ (Cat. #558106, Clone 53–6.7), 7AAD (Cat. #51-68981E), and Annexin-V (Cat. #550474) were purchased from BD Biosciences (Franklin Lake, NJ); and anti-CD4+^−^ was purchased from Tonbo Biosciences (Cat. #60–0042, Clone RM4–5, San Diego, CA). Cytokine multiplex was purchased from R&D Systems (Cat. #LXSAMSM).

### Quantitative real-time RT-PCR

Total RNA was extracted from sorted Vβ3 and Vβ14 CD4^+^ T cells using the miRNeasy mini kit (Qiagen, Valencia, CA #217004) and reverse-transcribed with an iScript cDNA synthesis kit (Bio-Rad, Hercules, CA #1708891). Real-time quantitative PCR measurement of cDNA was then performed using SsoAdvanced Universal SYBR^®^ Green Supermix (Bio-Rad, Hercules, CA #1725274) and a CFX96 real-time PCR instrument (Bio-Rad, Hercules, CA). Samples were run in duplicate and gene expression levels were normalized to β-actin. Relative mRNA expression was calculated using the 2^−ΔΔCt^ method (19).

### miR-150 qPCR methods

RNA was extracted from purified CD4^+^ and CD8^+^ T cells using Qiagen miRNeasy Micro kits (Qiagen cat. 217084) according to the manufacturer’s instructions. cDNA libraries were generated using TaqMan MicroRNA Reverse Transcription Kits (ThermoFisher cat. 4366596) according to the manufacturer’s protocol except with an RT incubation time of 40 minutes. Primers from ThermoFisher hsa-miR-150 and snoRNA202 TaqMan assays were used for cDNA generation (ThermoFisher cat. 4427975, Assay IDs 000473 and 001232). SnoRNA202, is a widely used and accepted endogenous control, as a reference gene for stability in expression across T cell lineages. SnoRNA202 has been used to control for miRNA expression in In CD8^+^ T cells (20, 21) and in CD4^+^ T cells (22, 23). The same ThermoFisher assays were used for RT-qPCR according to the manufacturer’s protocol. snoRNA202 was used as a reference gene for the assay. ΔCt was calculated by subtracting snoRNA202 Ct values from miR-150 Ct values, and represented as log fold change graphically as 2^-ΔCt^. In the experiments with SEA stimulation, ΔΔCt was calculated as ΔCt_SEA_ – ΔCt_naive_ for each sample and represented graphically as 2^-ΔΔCt^.

### RNA-seq

Bulk RNA-seq: For each group, 10^4^-10^6^ cells were lysed in Trizol lysis buffer. RNA was extracted and processed using a QIAGEN miRNEasy Extraction kit. RNA library construction was done using a SMART-Seq v.4 Ultra-low input kit (Takara Bio, Shiga, Japan) and cDNA was converted to sequencing library using NexteraXT DNA Library Prep Kit with indexing primers (Illumina, San Diego, CA). For transcriptomics, libraries were sequenced for single end 1x100bp reads at 30 million reads/sample on a NOVASeq 6000 (Illumina). Quality controlled reads (fastq) were aligned to mouse genome (mm10) using HISAT2 and BAM file conversion, sorting, and indexing were done with Samtools (24, 25). Read counts were obtained from resulting BAM files using Stringtie (26). For transcriptomics analysis, differentially expressed genes were identified using DESeq2 (FDR<.05) (27). FPKM values from Stringtie of most differentially expressed transcripts by FDR were used to create heatmaps of significant DEGs. Pathway analysis was conducted using PathfindR (28). Library preparation and sequencing was conducted by the Whitehead Institute Genome Technology Core (Boston, MA).

### Flow cytometry

Flow cytometry was performed as described before (29). For intracellular cytokine staining, cells were seeded in 96-well round-bottom plates and stimulated for 4-5 h with media alone or PMA (50 ng/ml; Calbiochem, Darmstadt, Germany) plus ionomycin (1 µg/ml; Invitrogen) in the presence of GolgiPlug (BD Biosciences). Cells were surface stained, fixed with 1.5% PFA, permeabilized with 1% Saponin, stained at 4°C, and analyzed by flow cytometry. Acquisition was performed by LSRIIa. All flow cytometry data were analyzed with FlowJo (Tree Star, Ashland, OR). For apoptotic assay, cells were seeded in 96-well round-bottom plates at either 37°C +5% CO2, or 4°C for 4 h. Cells were surface stained, then incubated in manufacturer-recommended buffer with Annexin-V and 7-AAD for 15 min and immediately analyzed by flow cytometry.

### Immunization

Mice were injected i.p. with 1 μg of *S. aureus* enterotoxin A diluted in 200 μl of BSS, or BSS alone.

### Cell culture

Cells were cultured for the indicated time in figure legends, at 37°C and 5% CO_2_ in 200 μl complete tumor medium (CTM), consisting of modified Eagle’s medium with 5% fetal bovine serum, amino acids, salts, and antibiotics.

### Mito stress test assay and Mitoflow

Naïve CD4^+^ T cells from spleen and lymph nodes (inguinal, brachial, axillary, and cervical) of C57BL/6J mice (6–9 weeks old) were purified by negative selection using Dynabeads™ Untouched™ Mouse Cells Kits from ThermoFisher Scientific (Waltham, MA #11415D). Cells were then differentiated *in vitro* using Dynabeads™ Mouse T-Activator CD3/CD28 for T cell Expansion and Activation from ThermoFisher Scientific (1:1 ratio) (Waltham, MA $11452D), plus human rIL-2 (30 U/ml) for 44-66 h. Purified CD4^+^ T cells (3 × 10^5^/well) were plated in 96-well Seahorse plates (Seahorse Bioscience, North Billerica, MA) previously coated with Cell-Tak (Corning, #354240). Cells were pre-treated with vehicle or sodium diethyldithiocarbamate trihydrate (DTC) (Sigma #228680) for 30 min (30). Oxygen consumption rates (OCR) were measured using an XF-96 extracellular flux analyzer and a Mito stress test kit as per the manufacturer’s instructions (Agilent, Santa Clara, CA #103015-100). As for Mitoflow assay, splenocytes from WT and miR-150-KO mice immunized with SEA for 48 h were isolated and left in culture (0.2x10^6^ cells/well in a 96-well plate) for 4 h. Mitoflow staining (Cell technology #FLO200-2) was added for the last 30 min of culture as per the manufacturer’s instructions. Surface staining was added for the last 5 min of staining (9 μl, antibodies are diluted directly in Fc block).

### Apoptosis assay and DTC induced cell apoptosis

Splenocytes from WT and miR-150 KO mice previously immunized with vehicle or SEA for 48 h were isolated and treated with 0, 10, or 100 μM of DTC (Sigma #228680) for 4 h. The apoptotic rate was calculated by gating Annexin V/7AAD double positive CD4+ and CD8+ T cells. Values are shown as a ratio between DTC-treated and vehicle-treated cells.

### Intracellular p-S6 staining

Splenocytes from WT and miR-150-KO mice previously immunized with vehicle or SEA for 48 h were isolated and single cell suspensions were washed, resuspended in FACS buffer (HBSS, 0.1% sodium azide and 3% fetal calf serum), kept on ice, and treated with FcR blocking solution for 30 min at 4°C with mAbs: α-CD8, α-CD4, α-Vβ3, and α-Vβ14. Live cells were gated using LIVE/DEAD™ Fixable Blue Dead Cell Stain Kit from ThermoFisher Scientific (Waltham, MA). For phosphoS6 (pS6) detection, cells were washed with FACS buffer and further processed for intracellular staining using Foxp3 fixation/permeabilization buffer (eBioscience, Waltham, MA #00-5521-00) as per the manufacturer’s instructions. The fixed and permeabilized cells were then incubated with the pS6 (Ser235, Ser236) antibody from ThermoFisher Scientific (Waltham, MA #12-9007-42) and diluted in 1x permeabilization buffer for 2 h at 4°C.

### Statistical analysis

A two-tailed Student’s unpaired test and ANOVA were used for data analysis, with values of p<0.05 (*) used as significant threshold; p< 0.01 is indicated as (**) and p<0.001 (***). All statistical analyses were performed using Prism-GraphPad (La Jolla, CA).

## Results

**Figure 1:**
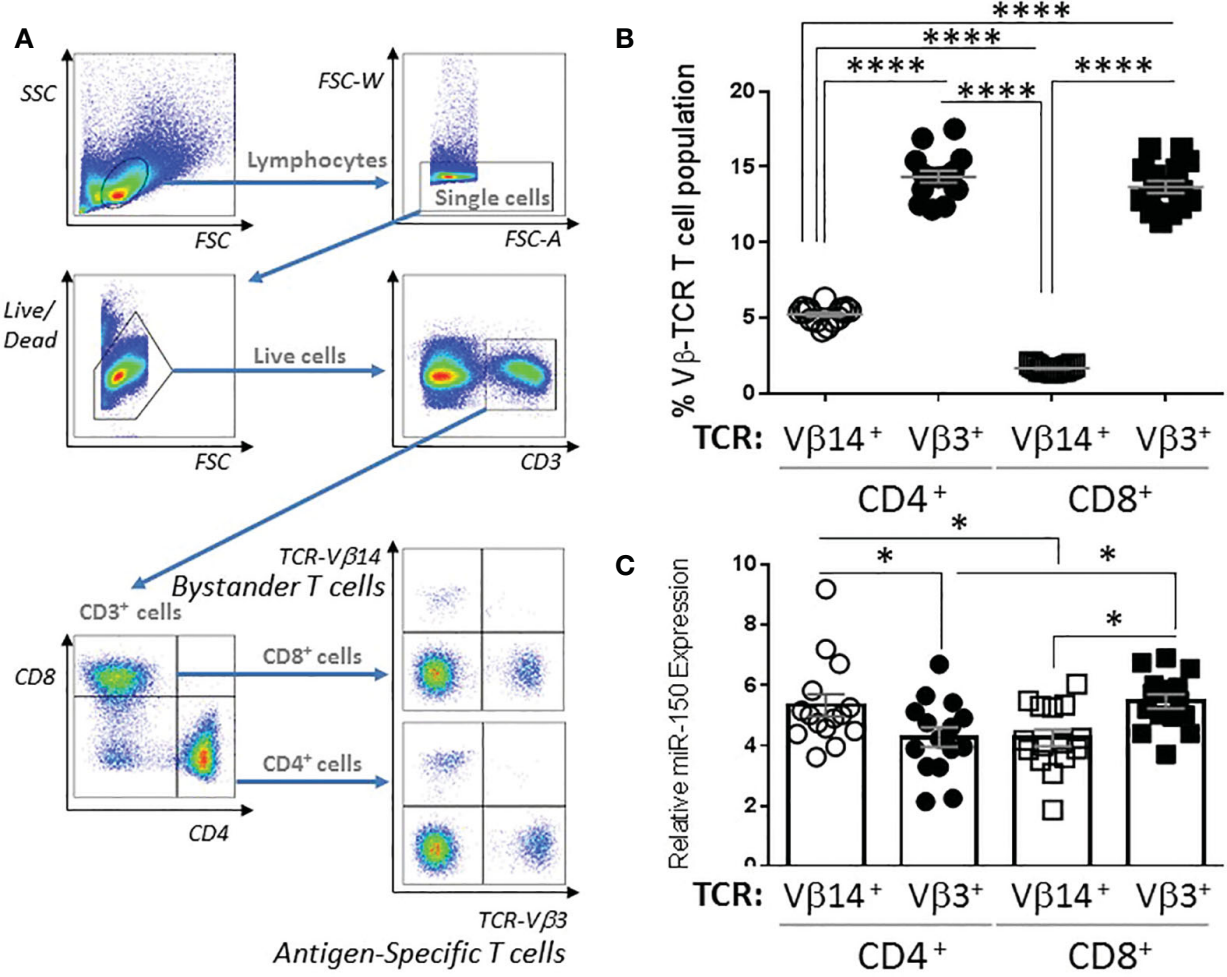
miR-150 expression is differentially regulated in CD4^+^ and CD8^+^ T cells. **(A)** Gating strategy of splenocytes from C57BL/6 mice 48 h post *S. aureus* enterotoxin A (SEA) injection. Cells were gated as lymphocytes based on FSC/SSC, single, live, CD3^+^, CD4^+^ or CD8^+^, and TCR-Vβ3^+^ or TCR-Vβ14^+^. **(B)** T cells were analyzed as described in A and reported as percent of the total CD4^+^ or CD8^+^ population (top panel). Data were combined from 2 independent experiments. Each dot represents a mouse, n=15/group. **(C)** T cells described in **B** were sorted to purity ~ 95-98%, RNA was obtained and miR-150 expression was reported for each cell population. Each dot represents a mouse, n=15/group +/- s.e.m. Statistical significance was evaluated by 2-way ANOVA. (ns: non-significant, (*p < 0.05; ****p < 0.0001).

miR-150 is highly expressed in CD8^+^ and CD4^+^ T cells; however, miR-150 has mostly been studied using transgenic systems where only one lineage is tested at a time, and mainly for CD8+ responses. To fully characterize miR-150 regulated T cell compartment in response to bacterial superantigen, we performed a thorough examination for cellular response *in vivo* and *ex vivo*. We immunized mice with the bacterial superantigen, staphylococcal enterotoxin A (SEA) that activates both endogenous antigen-specific CD8^+^ and CD4^+^ T cells with high frequency (**Supplementary Figure 1**). Subsequently, we observed strong response of endogenous SEA antigen-specific T cells (expressing T cell receptor (TCR)-Vβ3) and compared to non-antigen-specific T cells (also called bystander T cells; expressing TCR-Vβ14) in the same animal (**Figure 1A**). At 48 h post-SEA injection, antigen-specific TCR-Vβ3^+^ CD4^+^ and CD8^+^ T cell proportions were 2-3 fold higher than bystander TCR-Vβ14^+^ CD4^+^ and CD8^+^ populations (**Figure 1B**). Interestingly, antigen-specific CD4^+^ T cells displayed lower expression of miR-150 levels than bystander CD4^+^ T cells, whereas the reverse was observed in CD8^+^ T cells albeit higher proportion of antigen-specific T cells, miR-150 levels were slightly higher compared to bystander CD8+ T cells (**Figure 1C**).

**Figure 2:**
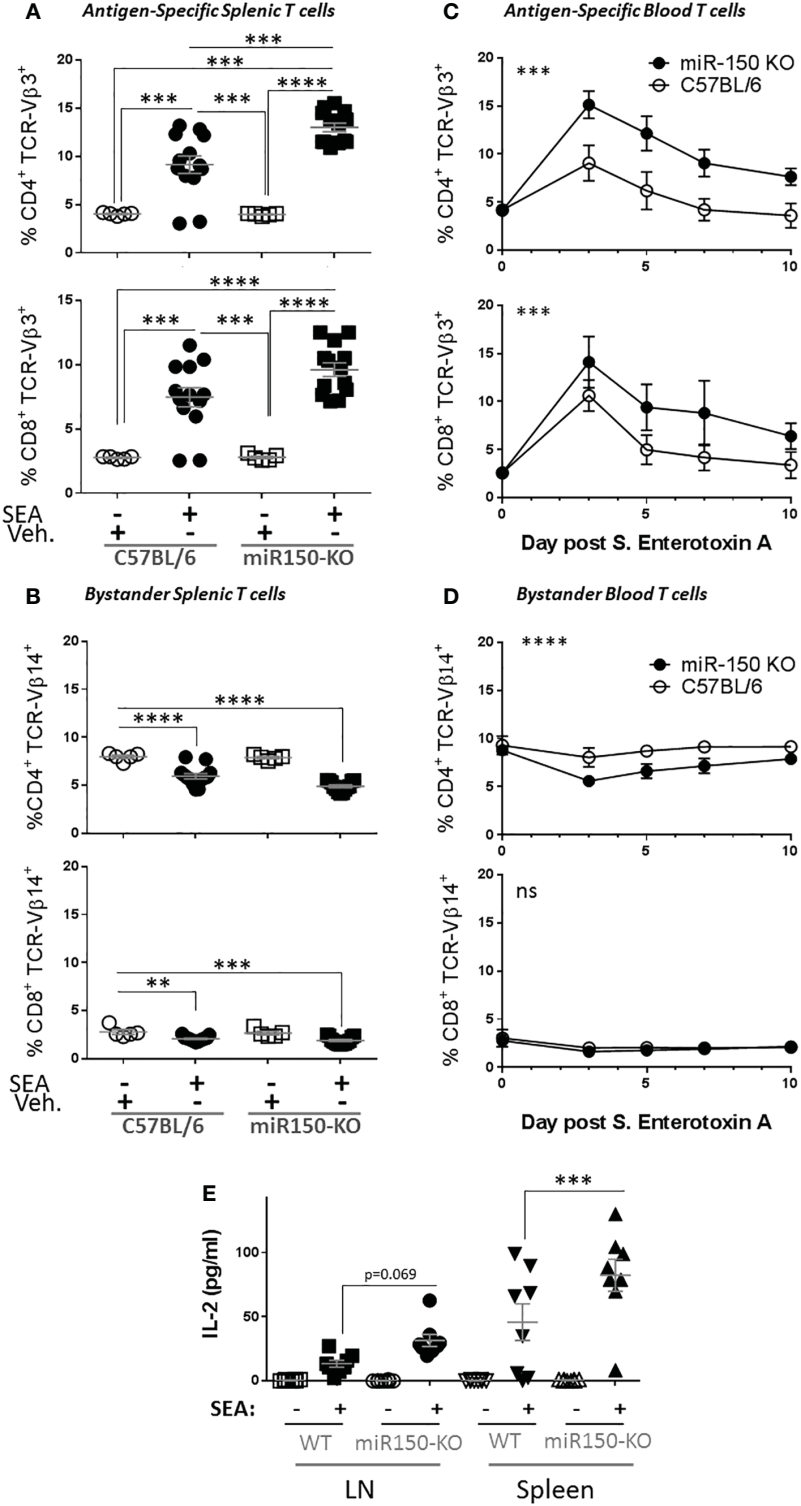
miR-150 controls T cell expansion after immunization. **(A)** Splenocytes from wild type (WT; C57BL/6) and miR-150-KO mice were analyzed by flow cytometry 48 h post-immunization with *S. aureus* enterotoxin A (SEA) or vehicle (Veh.) as described in **Figure 1A**. Percentage of antigen-specific (TCR-Vβ3) CD4^+^ (top row) and CD8^+^ (bottom row) T cells are shown. **(B)** Percentage of bystander (TCR-Vβ14) CD4^+^ (top row) and CD8^+^ (bottom row) T cells are shown. Data shown are individual biological replicates from 3 independent experiments +/- s.e.m. Statistical significance was evaluated by one-way ANOVA student’s t-test. **(C)** Mice immunized with SEA were bled every 2 or 3 days, and blood cells were analyzed as described in **Figure 1A**. Percentage of antigen-specific (TCR-Vβ3) CD4^+^ (top row) and CD8^+^ (bottom row) T cells are shown. **(D)** Percentage of bystander (TCR-Vβ14) CD4^+^ (top row) and CD8^+^ (bottom row) T cells are shown. Each line represents the average from 8 mice from 2 independent experiments. Statistical significance was evaluated by two-way ANOVA. **(E)** 10-days post-SEA immunization, lymph node cells (four left panels) and splenocytes (four right panels) from miR-150 and WT mice were re-stimulated with SEA or vehicle, culture supernatant was obtained after 18 h and analyzed by multiplex ELISA for 17 analytes (**Supplementary Figure 1**). Secretion of IL-2 is shown. Each dot represents an independent biological replicate from 2 independent experiments. Data shown are individual biological replicates +/- s.e.m. Statistical significance was evaluated by 1 way ANOVA. (ns: non-significant, **p < 0.01, ***p < 0.001; ****p < 0.0001).

Since our data uncovered differential miR-150 expression levels in antigen-specific CD4^+^ and CD8^+^ T cells (**Figure 1**), we investigated if miR-150 controls effector T cell expansion post stimulation using SEA-immunized miR-150-KO mice. Indeed, 48 h post-SEA immunization, a greater expansion of both CD4^+^ and CD8^+^ antigen-specific T cells was observed in miR-150-KO than wild type (WT) mice (**Figure 2A**). Consistent with this observation it was shown that miR-150 deficiency only enhanced antigen-specific T cells (TCR-Vβ3^+^) but not bystander T cells (TCR-Vβ14^+^) (**Figure 2B**). To further validate the observation, we immunized mice with SEA in the presence of LPS, which models pathologies such as SIRS, ALI or sepsis (10). A greater accumulation of both antigen-specific CD4^+^ and CD8^+^ T cells in miR-150-KO than in WT mice 7 days post-immunization was observed (**Supplementary Figure 2**). Furthermore, the kinetics of the T cell response in SEA-immunized miR-150-KO and WT mice was followed by measuring blood T cell populations over a 10 day period. A fast and robust antigen-specific CD4^+^ and CD8^+^ T cell expansion, superior in miR-150-KO than WT mice, was observed after 3 days and remained high during the 10 day period (**Figure 2C**), but no expansion was observed in bystander T cells (**Figure 2D**). Declining of the overall antigen-specific CD4^+^ and CD8^+^ in miR-150-KO and WT mice after day 3 suggested a predominant role for miR-150 early in T cell response, resulting in a higher percentage of T cells at day 10 in miR-150-KO mice.

To analyze the outcomes of the stronger miR-150-KO response, splenocytes from these mice were re-stimulated *in vitro* with SEA or vehicle. Culture supernatant was obtained after 18 h and analyzed by multiplex ELISA for 17 analytes (**Supplementary Figure 3**). As expected, SEA induced secretion of several cytokines and chemokines from both miR-150-KO and WT cells. Among them, IL-2 was the only cytokine with significantly higher release from miR-150-KO than WT cells (**Figure 2E**).

**Figure 3:**
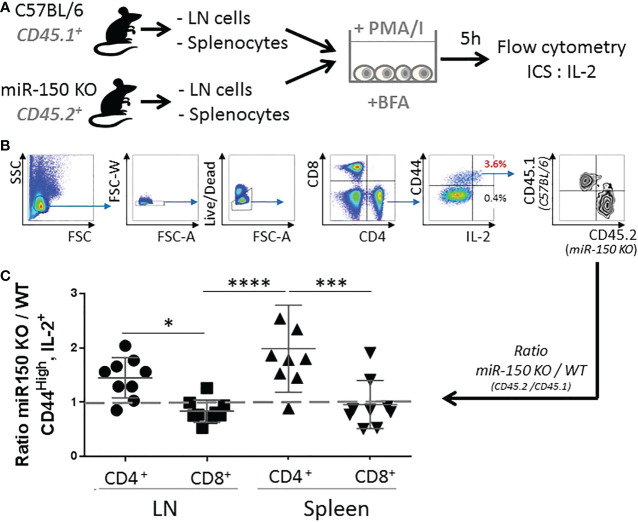
miR-150 controls IL-2 production in CD4^+^ but not CD8^+^ T cells. **(A)** Naïve splenocytes and naïve lymph node cells from miR-150-KO (CD45.2^+^) and WT (CD45.1^+^) mice were co-cultured and stimulated with PMA and Ionomycin (PMA/I) for 5 h in the presence of Brefeldin A (BFA) then analyzed by flow cytometry. **(B)** Cells were analyzed by flow cytometry and gated as single, live, CD4^+^ or CD8^+^, CD44^+^, and IL-2^+^ cells. **(C)** Ratio of miR-150-KO/WT of activated cells expressing IL-2 is shown. Data were combined from 3 independent experiments, n=9 per group. Each dot represent individual biological replicates +/- s.e.m. Statistical significance was evaluated by two-way ANOVA. (*p < 0.05, ***p < 0.001; ****p < 0.0001).

While the data showed higher overall secretion of IL-2, it was possible that higher levels of IL-2 in miR-150KO could be attributed to more cells resulting from cell expansion or increased IL-2 production on a per cell basis in antigen-specific T cells. Isolated naïve splenocytes and lymph node cells from WT (CD45.1^+^) and miR-150-KO (CD45.2^+^) mice were co-cultured with PMA and Ionomycin *in vitro* for 5 h (**Figure 3A**). Intracellular IL-2 levels were measured by flow cytometry on a per cell basis using congenic markers to identify cell populations in the co-culture (**Figure 3B**). This method was developed to enable comparison of proportional contribution of antigen-responding cells from different tissues under identical stimulation condition. Flow analysis showed that activated CD4^+^ T cells from the lymph nodes and spleen expressing IL-2 were, respectively, 1.5 and 2 times more numerous in miR-150-KO than WT mice (**Figure 3C** first and third groups). However, the IL-2 expressing cell ratio of miR-150-KO *vs*. WT was ~1 for CD8^+^ T cells from the lymph nodes and spleen (**Figure 3C** second and fourth groups), demonstrating a clear distinction that miR-150 limits the ability of CD4^+^ T cells, but not CD8^+^ T to produce IL-2.

**Figure 4:**
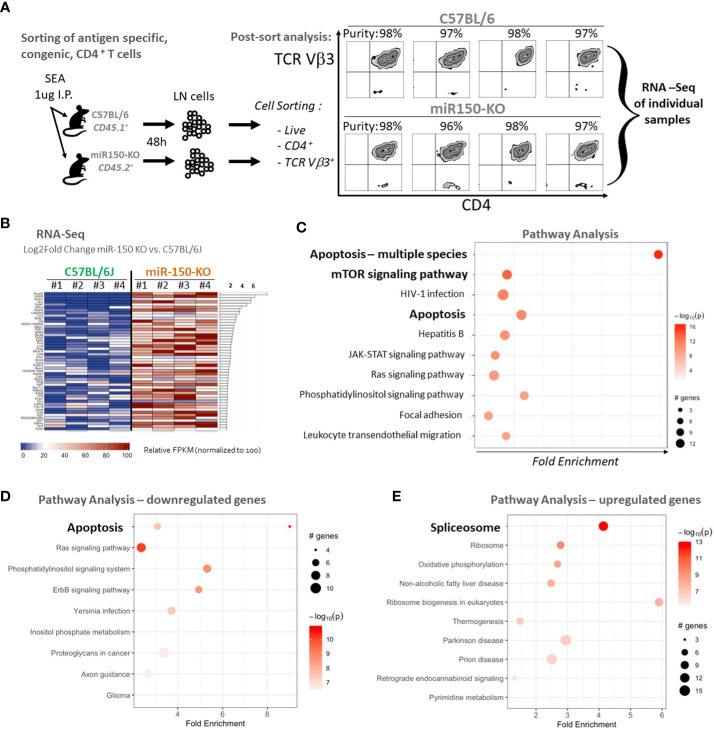
Apoptotic pathways are under regulation of miR-150 in antigen-specific CD4^+^ T cells. **(A)** Lymph node (LN) cells from miR-150-KO and WT mice were stained 48 h post-SEA challenge with lineage-specific antibodies, and sorted to high purity. **(B)** Pathway analysis with PathfindR was conducted on 674 differentially expressed genes using both upregulated (390 genes) and downregulated (284 genes) genes filtered from DESeq using a p-value differential cutoff of p-value<.05. Relative FPKM levels of the top 50 (of 674 genes) ranked by log_2_fold change between C57BL/6J and miR-150-KO sorted CD4^+^ Vb3^+^ cells (FDR<.05) are shown, n=4/group. **(C)** Pathway analysis of 674 genes identified in **4B** ranked by log2fold change between C57BL/6J and miR-150-KO n=4 per group. **(D)** Pathway analysis of 284 genes found downregulated in miR150 KO vs WT CD4+ T cells by log2Fold Change with p-value <.05. n=4 per group. Two distinct but overlapping KEGG pathways (mmu04210 and mmu04215) for Apoptosis were identified by PathfindR and as such, appear as two circles on the same line. **(E)** Pathway analysis of 390 genes found upregulated in miR150 KO vs WT CD4+ T cells by log2Fold Change with p-value <.05. n=4 per group. Canonical apoptotic regulators Bax, Bid, Cycs were grouped under alternative disease pathways (Non-alcoholic fatty liver disease and Prion disease).

To characterize how miR-150 programs antigen stimulation during the CD4^+^ T cell response, RNA-seq analysis of sorted antigen-specific CD4^+^ T cells post-SEA immunization in miR-150-KO and WT mice was performed lymph nodes cells from both miR-150-KO (CD45.2^+^) and WT mice 48 h post SEA immunization were, stained, and sorted for antigen-specific CD4^+^ T cells from each mouse using lineage markers (**Figure 4A**). RNA-seq analysis of purified antigen-specific CD4^+^ T cells compared the top genes ranked by log_2_ fold change between miR-150-KO vs. WT groups; 674 significantly differentially expressed genes were detected (FDR<.05) (**Figure 4B**). Apoptotic canonical gene signatures and mTOR signaling were the most significantly enriched pathways in miR-150 KO antigen-specific CD4^+^ T cells (**Figure 4C**). We specifically looked for changes in genes of the IL-2 signaling pathway but did not observed any significant changes (**Supplementary Table 2**). Analysis of only downregulated genes confirmed that genes involve in apoptosis could be regulated by miR-150 (**Figure 4D**). Interestingly, analysis of only upregulated genes revealed that splicosome is a pathway enriched in miR-150-KO CD4^+^ T cells, an observation supported by our recent finding that hyper activated T cells have upregulation of RNA binding proteins regulating splicing (**Figure 4E**) (31).

**Figure 5:**
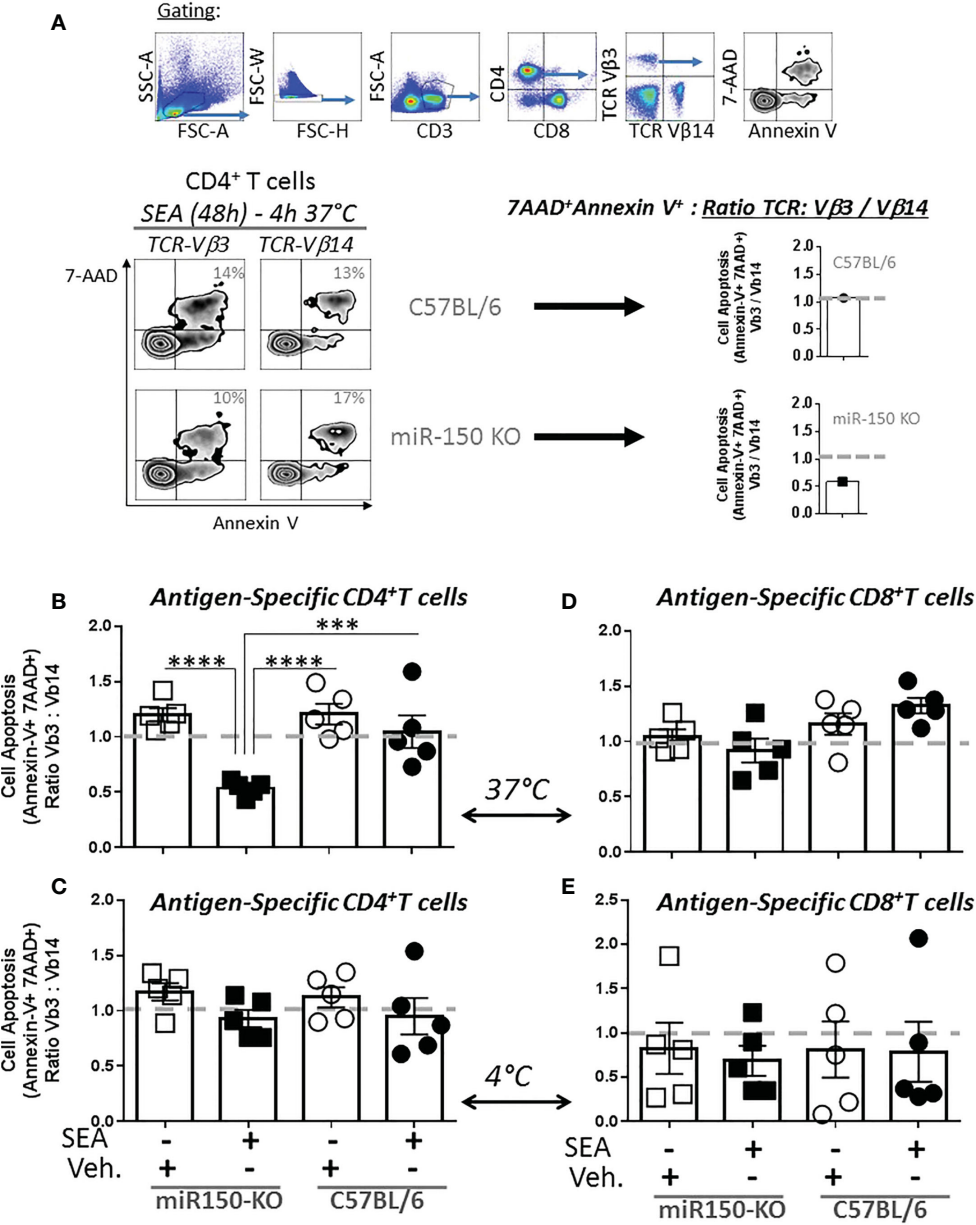
miR-150 regulates apoptosis of antigen-specific CD4^+^ T but not CD8^+^ T cells. **(A)** Splenocytes from miR-150-KO and WT mice analyzed 48h post-SEA and post-vehicle challenge and 4 h post-incubation *ex vivo* for expression of apoptotic markers (7AAD, Annexin V) and reported as a ratio of TCR: antigen-specific (Vβ3) *vs.* bystander (Vβ14). **(B)** Analysis of antigen-specific CD4^+^ T cells incubated for 4 h at 37°C or **(C)** bystander CD4+ T cells incubated for 4 h at 4°C is shown. **(D)** Analysis of antigen-specific CD8^+^ T cells incubated 4h at 37°C or **(E)** bystander CD8+ T cells incubated 4h at 4°C is shown. Each dot represents a biological replicate, five independent experiments are shown +/- s.e.m. Statistical significance was evaluated by 2 way ANOVA. (***p < 0.001; ****p < 0.0001).

To functionally validate the role of miR150 in controlling apoptosis, we measured apoptotic potential of T cells 48 h post-SEA immunization by flow cytometry (**Figure 5A**, top panel). Since apoptotic T cells are rapidly cleared *in vivo*, apoptosis was measured *ex vivo* for 4 h at 37°C (**Figure 5A**, left panel). We report the proportion of apoptotic cells with Annexin-V and 7-AAD staining paired comparison between antigen-specific (TCR-Vβ3^+^) and bystander (TCR-Vβ14^+^) T cells from the same mouse. A TCR-Vβ3^+^/Vβ14^+^ ratio of 1 indicates a similar level of apoptosis in antigen-specific and bystander CD4^+^ or CD8^+^ T cells whereas a lower ratio indicates reduced apoptosis in antigen-specific T cells (**Figure 5A**, right panel). In the absence of immunization, TCR-Vβ3^+^ and TCR-Vβ14^+^ CD4^+^ T cells were at an equivalent level of apoptosis (**Figure 5B**, first and third column). On the contrary, after immunization, antigen–specific CD4^+^ T cells (TCR-Vβ3^+^) were significantly more resistant to apoptosis than bystander T cells (TCR-Vβ14^+^) in miR-150-KO, while the apoptosis ratio in WT cells was ~1 (**Figure 5B**, second and fourth column). Importantly, apoptosis analysis of the same cells placed for at 4°C for 4 h (to impede metabolic function*)* did not show any difference between antigen-specific and bystander CD4^+^ T cells (**Figure 5C**). The same control was performed on CD8^+^ T cells and showed no difference in apoptosis between antigen-specific and bystander T cells (**Figure 5D, E**, **Supplementary Table 1**), confirming a differential role for miR-150 in CD4^+^ vs. CD8^+^ T cells in response to SEA stimulation. The addition of IL-2 during the 4 h apoptotic assay were similar as when exogenous IL-2 was absent (**Supplementary Figure 4**), suggesting that T cells, already programmed for activation-induced cell death *in-vivo*, were not rescued by IL-2.

**Figure 6:**
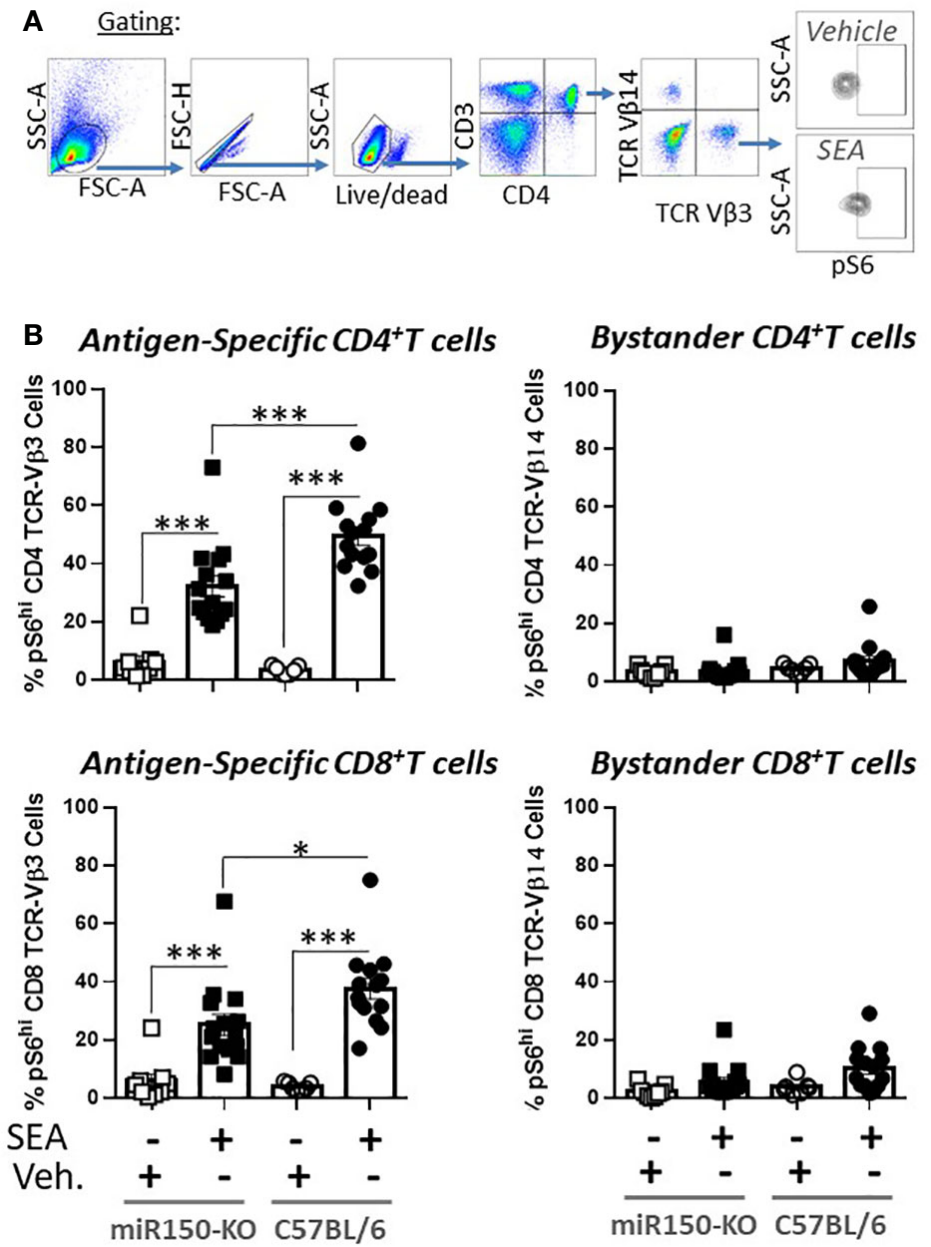
miR-150 regulates pS6 antigen-specific T cells. **(A)** Splenocytes from miR-150-KO and WT mice analyzed 48 h post-SEA and post-vehicle challenge and 4 h post-incubation *ex-vivo* for expression of mTOR marker pS6. **(B)** Analysis of CD4^+^ and CD8^+^ T cells. Each dot represents a biological replicate from 4 independent experiments are shown +/- s.e.m. Statistical significance was evaluated by one way ANOVA. (***p < 0.001, ****p < 0.0001).

**Figure 7:**
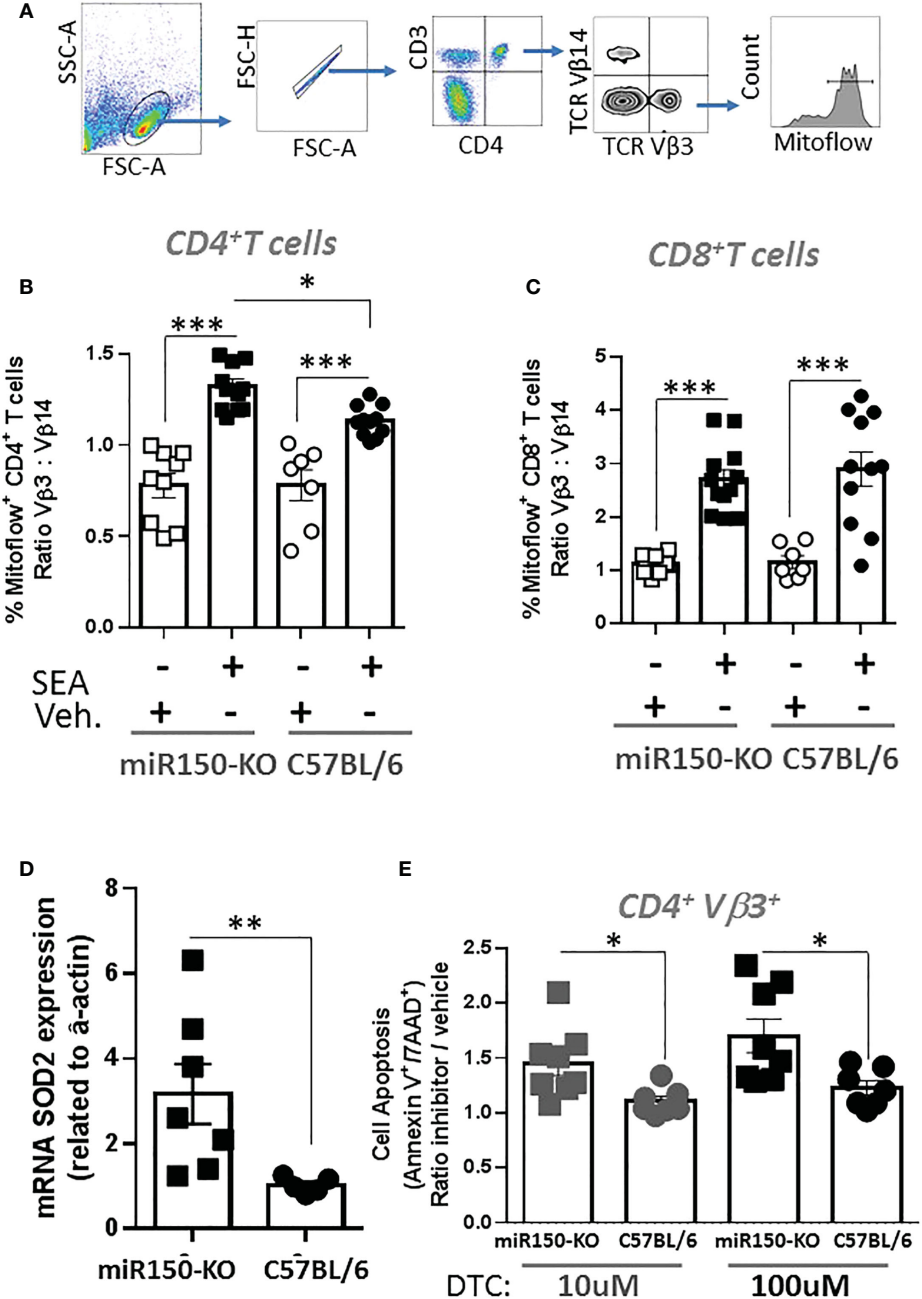
miR-150 controls superoxide-dependent apoptosis of antigen-specific CD4^+^ T cells. **(A)** Splenocytes from miR-150-KO and WT mice analyzed 48 h post-SEA or -vehicle challenge and 4 h post-incubation *ex vivo* for expression of mitoflow, an indicator of mitochondrial membrane potential. **(B)** Analysis of CD4^+^ T cells, and **(C)** CD8^+^ T cells mitoflow staining is reported as a ratio of TCR: Vβ3 *vs.* Vβ14. Each dot represents a biological replicate from 3 independent experiments are shown +/- s.e.m. Statistical significance was evaluated by one way ANOVA. **(D)** mRNA SOD2 expression vs. actin from sorted antigen-specific CD4^+^ T cells is reported. Each dot represents a biological replicate from 2 independent experiments are shown +/- s.e.m. Statistical significance was evaluated by student’s t-test. **(E)** Analysis of antigen-specific CD4^+^ T cells incubated 4 h at 37°C in presence of the SOD2 inhibitor DTC. Apoptosis is reported as ratio inhibitor vs. vehicle as described and gated in **Figure 5A**. Each dot represents a biological replicate, three independent experiments are shown +/- s.e.m. Statistical significance was evaluated by one way ANOVA. (*p < 0.05, **p < 0.01, ***p < 0.001).

The impact of miR-150 deficiency on the mTOR pathway (**Figure 4C**), was assessed 48 h post-SEA immunization by measuring S6 phosphorylation (pS6), a precise marker of mTORC1 activity (32, 33) (**Figure 6A**). Expression of pS6 increased after immunization in antigen-specific T cells only, but more strongly in WT than miR-150-KO in both CD4^+^ and CD8^+^ T cells (**Figure 6B**). As reduced mTOR activity has been shown to prevent CD4^+^ T cell apoptosis (34), it was reasoned that the mitochondria should have greater viability in miR-150-KO than WT antigen-specific CD4^+^ T cells. Hence, mitochondrial membrane potential was measured using Mitoflow (**Figure 7A**), which showed stronger membrane potential when comparing the ratio of antigen-specific vs. bystander CD4^+^ T cells from miR-150-KO over WT mice (**Figure 7B** and **Supplementary Table 3**), but again no difference was observed between miR-150-KO and WT in CD8^+^ T cells (**Figure 7C**).

To identify genes involved in the mechanism of mitochondrial function and mitosis, the RNA-seq data was mined from sorted antigen-specific CD4^+^ T cells post-SEA treatment (**Figure 4**). Of the 674 genes significantly different (FDR<.05) between miR-150-KO and WT groups, we found 134 genes with predicted miR-150 targets from two databases (**Supplementary Figure 5**). Among them, 66 genes were upregulated in miR-150-KO antigen-specific CD4^+^ T cells and 4 genes were known to participate in reducing superoxide byproducts that are pro-apoptotic. Both superoxidedismutase-2 (SOD2) and netrin-1 (Ntn1) directly reduce superoxide products (35), while Nfe2l2 and Frataxin (Fxn) enhance and promote SOD2 function (36, 37). Therefore, either directly or indirectly, miR-150 may control superoxide levels in antigen-specific CD4^+^ T cells. SOD2 mRNA expression was measured in miR-150-KO antigen-specific CD4^+^ T cells and was validated to be higher in miR-150-KO than WT cells (**Figure 7D**). Treatment with sodium diethyldithiocarbamate trihydrate (DTC), a SOD2 inhibitor (30, 38), 48 h post-SEA immunization (in the same apoptosis assay as performed in **Figure 5**), demonstrated a titratable effect in miR-150-KO antigen-specific CD4^+^ T cells that was more sensitive than in WT CD4^+^ T cells, confirming a role for the production of superoxide products in T cell apoptosis (**Figure 7E**). No difference was observed in antigen-specific CD8^+^ T cells or bystander CD4^+^ T cells (**Supplementary Figure 6**).

## Discussion

A growing number of studies have shown that miR-150 has a general effect on T cell responses (6, 7, 17, 39–43); our work specifically demonstrates the direct impact of miR-150 on antigen-specific CD4^+^ and CD8^+^ T cells *in vivo* while carefully controlling for effects on bystander T cells. Although miR-150 controls both CD4^+^ and CD8^+^ T cell expansion similarly, we show that it regulates IL-2 production and apoptosis in CD4^+^ T cells, thereby limiting their ability to help CD8^+^ antigen-specific T cells. Mechanistically, we identify that apoptosis is specifically regulated by miR-150 in antigen-specific CD4^+^ T cells through control of superoxide products. Thus, while classical studies have shown how CD4 T cells help through the action of cytokines like IL-2, costimulation and accessory signals, our research demonstrates a key pathway mediated by miR-150 that attenuates CD4 T helper activity.

The superantigen immunization instigates potent T cell responses *in vivo* that model ALI (8, 9), SIRS (10), and lethal toxic shock (13, 14). SEA directly binds MHC on antigen-presenting cells without processing and specifically cross-linking TCR-Vβ chains on CD4^+^ and CD8^+^ T cells. Thus, both CD4^+^ and CD8^+^ T cells are exposed to the same antigen *in vivo* and in concert mount antigen specific responses. Bypassing TCR peptide specificity has shown no known bias toward memory or naïve T cells (44), and activating a large population of SEA-specific T cells (~5% of total) induces a strong systemic oligoclonal T cell activation in a few hours followed by robust expansion (8). Importantly, this system allowed simultaneous measurement of miR-150 in antigen-specific CD4^+^ and CD8^+^ T cells along with bystander, inactivated T cells. Previous studies have shown miR-150 to be highly expressed in naïve CD4^+^ and CD8^+^ T cells and during T cell development (18, 39–41, 45). *In vitro* activation of CD4^+^ T cells induces downregulation of miR-150 (45–47) including in regulatory T cells (42), but this has not been confirmed *in vivo*. In our system, we measured a decrease of miR-150 in antigen-specific CD4^+^ T cells when compared with bystander CD4^+^ T cells. This comparison is critical to capture cells programmed for apoptosis *in vivo*. Previous miR-150 studies in CD8^+^ T cell fate determination uncovered a complex picture. MiR-150 was down-regulated following *in vivo a*ctivation of TCR transgenic CD8^+^ T cells (6, 7) and *in vitro* activation of human anti-HIV CD8^+^ T cells (48). However, similar to our study, miR-150 can also be up-regulated, especially in terminal effector CD8^+^ TCR transgenic T cells (7) and in CD8^+^ TCR transgenic T cells activated *in vitro* (17).

Genetic deletion of miR-150 in mice leads to an increase of both CD4^+^ and CD8^+^ T cell antigen-specific responses as early as 3 days post-immunization. Thus, miR-150 has a critical role in buffering the initial T cell response that could otherwise promote autoimmunity as observed in CD4^+^ T cells from Myasthenia Gravis patients (43). The increased secretion of IL-2 in miR-150-KO over all other cytokines tested as well as the preferential overproduction of IL-2 in miR-150-KO CD4^+^ T cells is congruent with a stronger initial expansion of T cells and miR-150 being a key regulator of T helper activity. Hence, early IL-2 overproduction by CD4^+^ but not CD8^+^ T cells benefits both CD4^+^ and CD8^+^ lineages to clonally expand. It is noteworthy that miR-150 has not (to our knowledge) been directly implicated in IL-2, IL-2 receptors, or STAT-5 mRNA regulation. Thus, it is fitting that exogenous IL-2 did not control T cell apoptosis in our apoptosis assay (**Supplementary Figure 4**), suggesting the cells were most likely programmed *in vivo* early during T cell priming, but additional experiments beyond the scope of this work would be required to firmly establish that possibility. Other studies have shown that miR-150 can promote CD4^+^ T cell apoptosis and impair IL-2 production *in-vitro* (49, 50), but our study demonstrates that miR-150 deletion in CD4^+^ T cells inhibited activation-induced cell death *in vivo*, which resulted enhanced clonal expansion. Secondly, our novel RNA-Seq/pathway analysis and *in vitro* work demonstrated a novel link between miR150 regulation of SOD2 impacting mitochondrial function and apoptosis. Moreover, to our knowledge we identified the first evidence of a functional link between miR-150 and the splisosome. Interestingly, the effects of ectopic miR-150 expression on CD4 T cell survival has been previously tested mainly *in-vitro* systems. Unfortunately, miR-150 overexpressed in hematopoietic stem cells and injected into lethally irradiated recipient mice only showed very slight increase of mir-150 in mature CD4 and CD8 T populations making this approach unpractical *in vivo* (41). However, ectopically expressed anti-miR-150 and miR-150 in CD4 cells derived from myasthenia gravis patients presented some *in-vitro* difference in survival with anti-miR-150 but not with miR-150 (43). However, miR-150 delivered by lentivirus into human CD4^+^ T cells promoted apoptosis (50). Interestingly miR-150, along other miRNAs, has been found to inhibit HIV messengers in resting CD4 T cells (47, 51). Therefore manipulating miR-150 expression in CD4 T cell could be used for therapeutic purposes.

The dominant enrichment of apoptosis pathways and the mTOR pathway revealed by RNA-seq suggested that miR-150 specifically affects the mitochondria of antigen-specific CD4^+^ T cells by down-regulating targets involved in survival/apoptosis (**Figure 4**). We validated this finding by showing that antigen-specific miR-150-KO CD4^+^ T cells are specifically less apoptotic (**Figure 5**) and have healthier mitochondria than WT cells (**Figure 6**, **7B, C**). MiRNA are known to block specific cellular functions by moderately down-regulating several components in the same pathway (52). A similar phenomenon may take place in our system where SOD2, along with SOD2-inducing genes Fxn and Nfe2l2 (**Supplementary Figure 5**), may be controlled by miR-150. SOD2 is a potent antioxidant enzyme, which binds to the superoxide anions to convert them to hydrogen peroxide and oxygen (53). An increase in SOD2 expression could be beneficial by decreasing reactive oxygen species production as it was described in CD4^+^ T cells during the acute phase of Trypanosoma cruzi infection early during T cell expansion (54). Similar to SOD3, SOD2 could also control the activation and differentiation of CD4+ T cells (55). From this study, we showed that miR-150 regulates SOD2 and both mouse and human SOD2 are listed on TargetScan as a possible direct targets for miR-150 but with only 1 putative 7mer binding site, suggesting a very weak interaction. Therefore, at this stage, we cannot conclude if the SOD2 contribution is from direct or indirect regulation of miR-150. Further direct molecular studies will be required to test the direct effect of mir-150 on SOD2 expression.

Importantly, SOD2 and NTn1 may not be the only genes involved in CD4^+^ T cell survival/apoptosis in our system. Our RNA-seq analysis pinpointed 23 other genes (predicted miR-150 targets and upregulated in miR-150-KO) with anti-apoptotic properties (**Supplementary Table 1**), suggesting miR-150 may regulate an array of mRNA to achieve the downregulation of multiple anti-apoptotic targets resulting in a multipronged apoptotic program.

Altogether, our work sheds new light on a T helper role for miR-150 in CD4^+^ T cells, especially early during the T cell response, by identifying specific regulation of apoptosis and control of superoxide products in antigen-specific CD4^+^ but not CD8^+^ T cells.

## Data availability statement

The datasets presented in this study can be found in online repositories. The names of the repository/repositories and accession number(s) can be found below:GSE216981 (GEO).

## Ethics statement

The animal study was reviewed and approved by University of Connecticut Health’s Animal Care Committee.

## Author contributions

AV and AM conceived and designed the study. AM, TK, and FA analyzed the data. AM drafted the manuscript. AM, FA, TK, and KK performed experiments. BZ provided reagents, experimental expertise, and reviewed the manuscript. All authors contributed to the article and approved the submitted version.
