# Supplementary figures and images for: Antigen-specific downregulation of miR-150 in CD4 T cells promotes cell survival
Source: Front Immunol. 2023 Jan 27;14:1102403. doi: 10.3389/fimmu.2023.1102403 (PMC9936563; doi:10.3389/fimmu.2023.1102403)

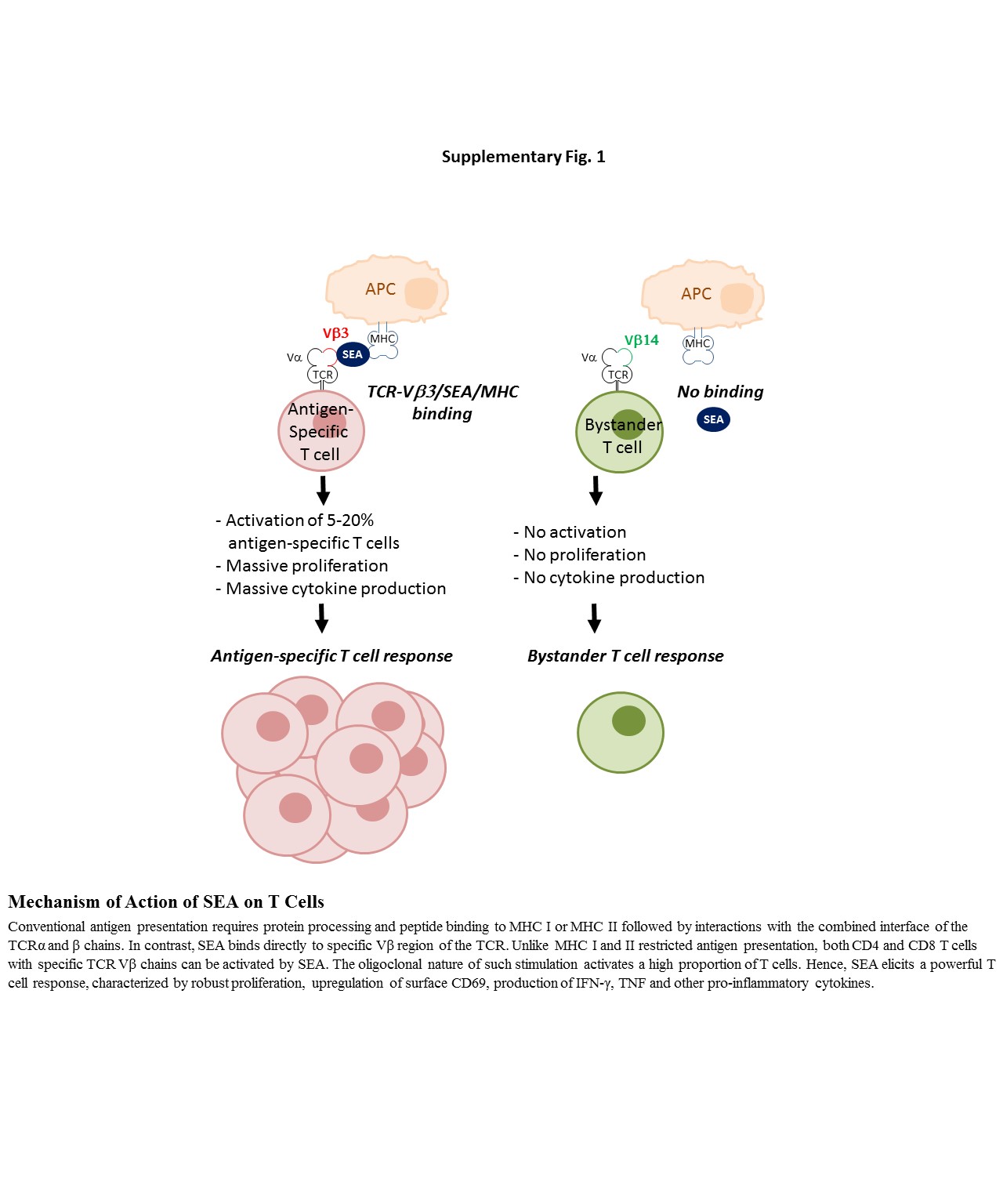

Supplement: Supplementary file 1 [file Image_1.jpeg]

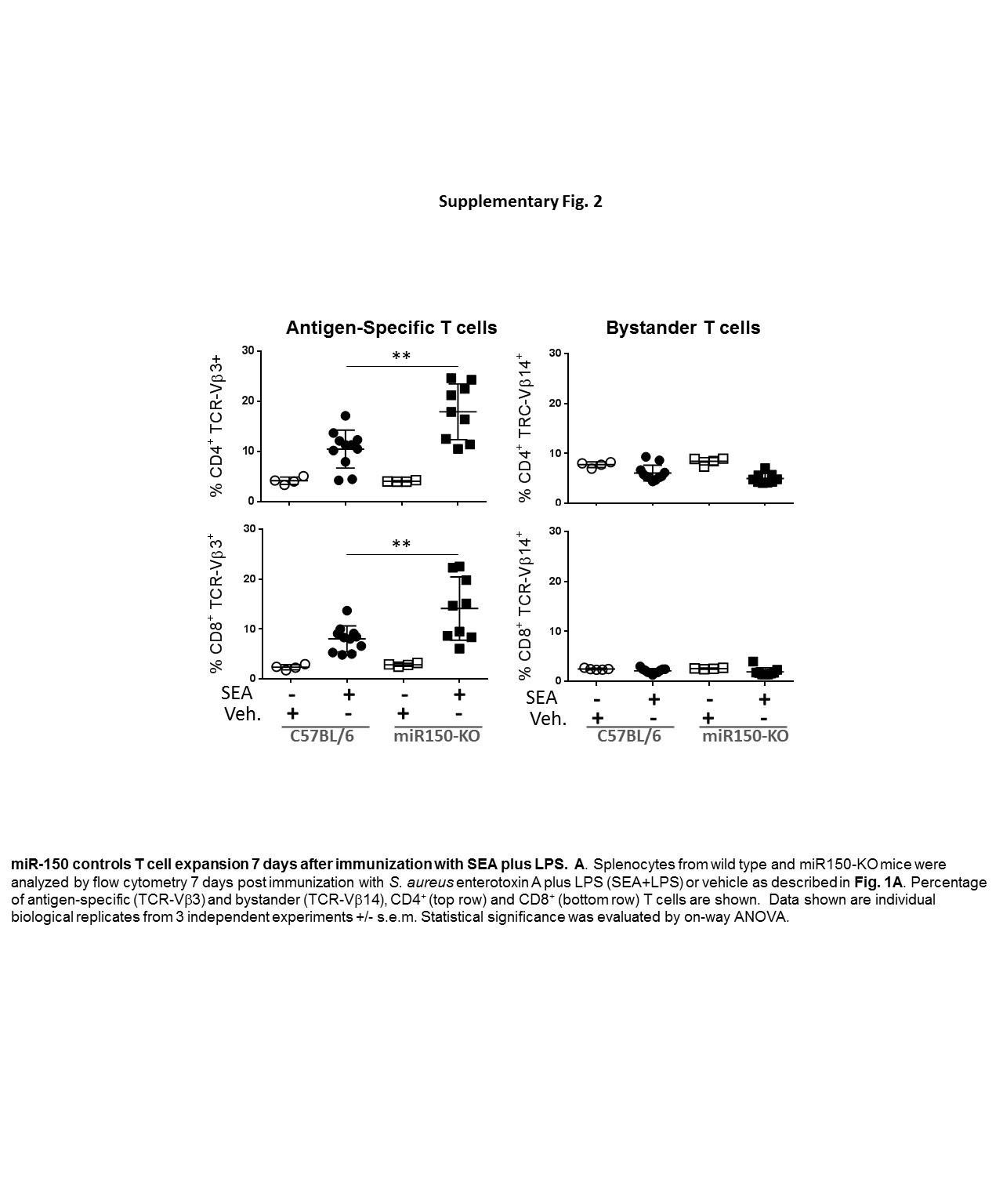

Supplement: Supplementary file 2 [file Image_2.jpeg]

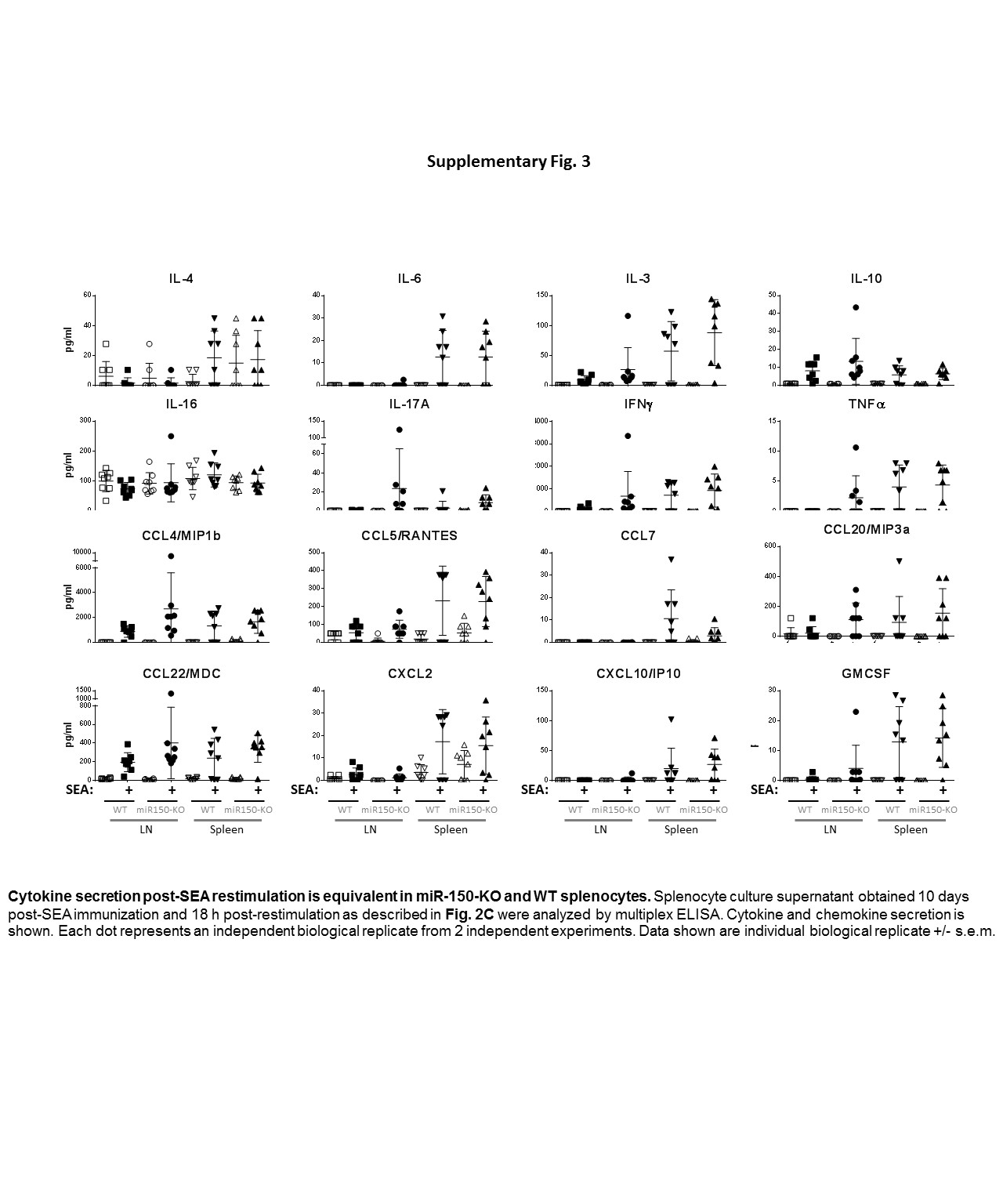

Supplement: Supplementary file 3 [file Image_3.jpeg]

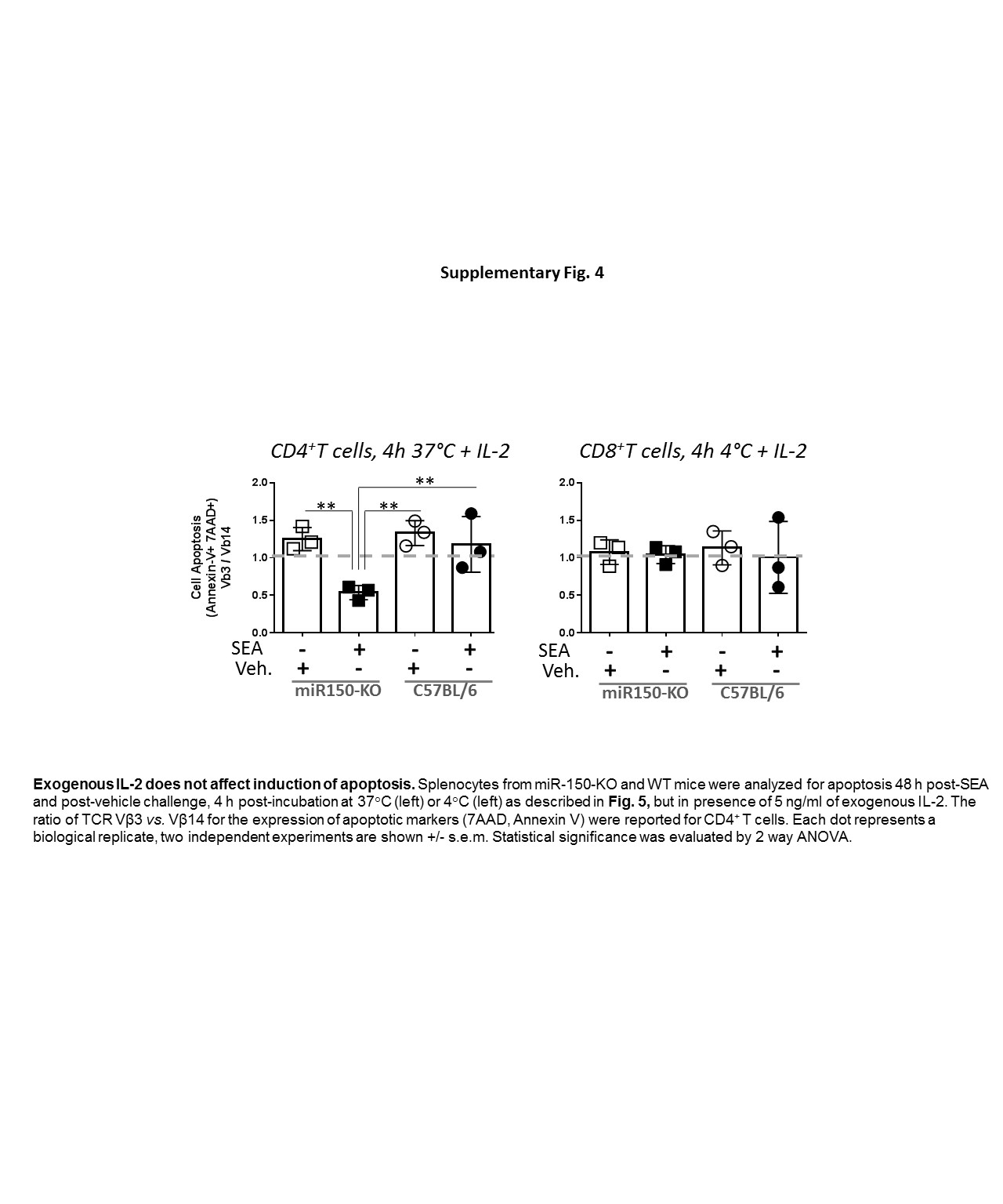

Supplement: Supplementary file 4 [file Image_4.jpeg]

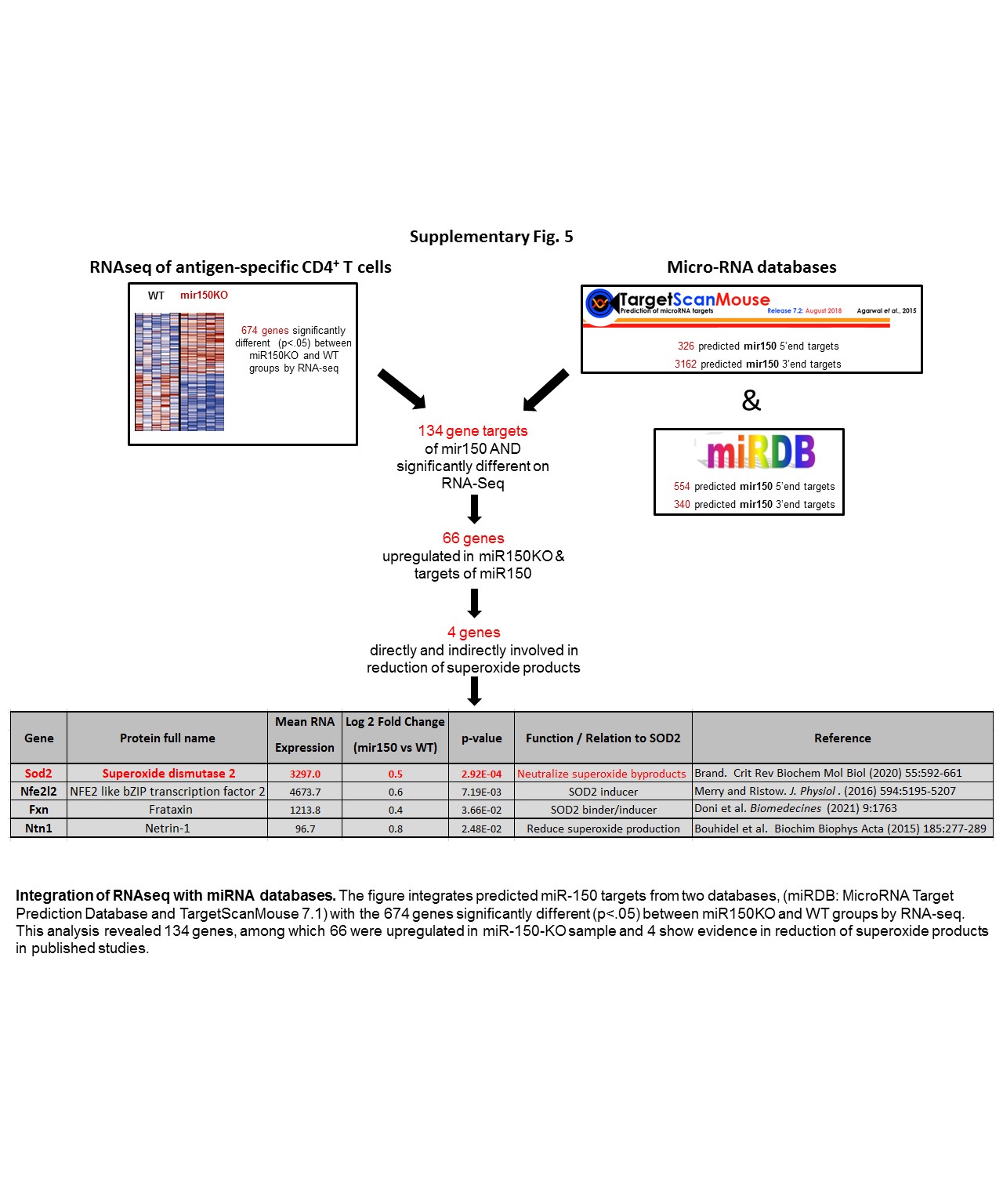

Supplement: Supplementary file 5 [file Image_5.jpeg]

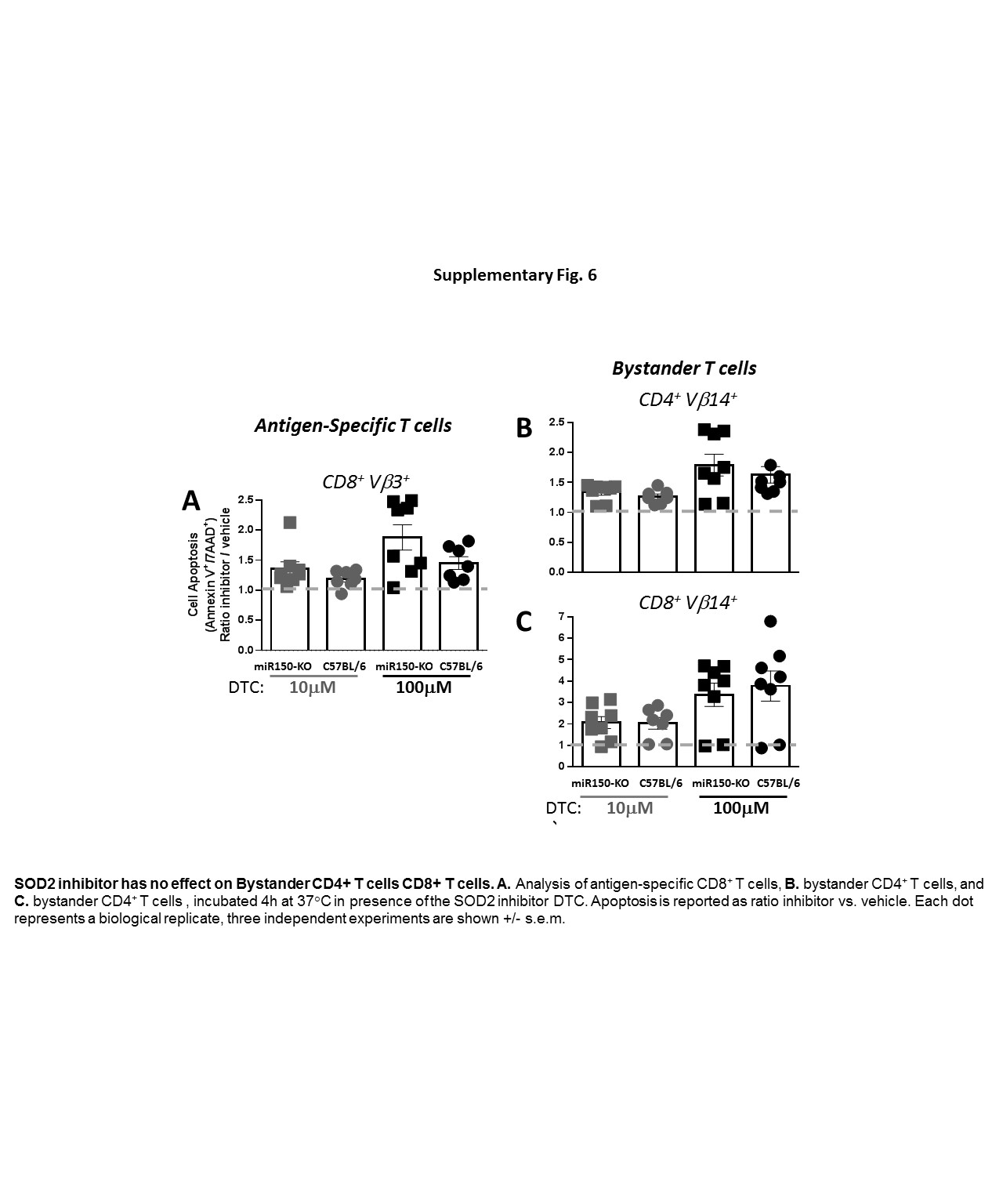

Supplement: Supplementary file 6 [file Image_6.jpeg]
